# Reply to A. Mangla

**DOI:** 10.1200/GO.23.00145

**Published:** 2023-07-13

**Authors:** Muhammad A. Mansha, Ahmed N. Abbasi, Muhammad M. Fareed

**Affiliations:** Muhammad A. Mansha, MBBS, FCPS, Department of Radiation Oncology, Dow University of Health Sciences, Karachi, Pakistan; Ahmed N. Abbasi, MBBS, FFR RCS, Section of Radiation Oncology, The Aga Khan University, Karachi, Pakistan; and Muhammad M. Fareed, MD, Department of Radiation Oncology, West Virginia University Cancer Institute, Morgantown, WV

We are very grateful to Mangla^1^ for taking keen interest in our study and highlighting the need of incorporating chemotherapy in selected patients using validated nomograms for the treatment of extremity soft tissue sarcomas (ESTSs) to achieve better outcomes.^2^ Second, they have enumerated many advantages of preoperative radiation therapy (RT) and advocated for neoadjuvant treatment approaches in the management of locally advanced ESTS.

The heterogeneity and rarity of ESTS pose a challenge in decision making and demand a distinct site-specific therapeutic multidisciplinary team (MDT) approach.^3^ Furthermore, we perform a robust peer review process in the department of radiation oncology to ensure the quality of MDT recommendations and radiation treatment.^4^

Wide surgical excision is the standard definitive treatment, with RT being used as an adjunct modality to enable effective surgical resections or to offset the effects of a positive margin. This combination leads to a local control rate of 85%-90% with good limb function. However, amputation is still performed in some patients with advanced disease, who are elderly and have multiple comorbidities.

Over the years, there has been a diverse opinion among the oncologists regarding the use of chemotherapy in ESTS, particularly in adults. Theoretically, chemotherapy provides a double advantage of making bulky and deep-seated tumors easily resectable, thereby providing good local control and also eradicates any distant micrometastases to decrease the risk of distant recurrences. With 26 of 40 patients in our study presenting with distant recurrences,^2^ there is a desperate need to incorporate any form of systemic treatment to curtail distant metastases, as both surgery and RT are local modalities. Although chemotherapy was given in 35 patients, patient selection needs to be reviewed.

A randomized trial comparing surgery alone and neoadjuvant chemotherapy followed by surgery did not show a major survival advantage for patients receiving preoperative chemotherapy alone.^5^ In another retrospective study, a benefit of neoadjuvant chemotherapy was demonstrated in patients with high-grade tumors larger than 10 cm.^6^ With such conflicting trial designs and results, the use of chemotherapy is still an ongoing debate.

The risk stratification using nomograms helps in providing personalized treatment strategies. Over the years, with the inclusion of new prognostic tools and validation on different populations, these nomograms aid clinicians in selecting patients suitable for intensive treatments.^7^ A randomized study conducted by various European sarcoma groups showed that patient selection for chemotherapy using nomograms results in an improvement in overall survival.^8^

Positive surgical margin is a strong predictor of local recurrence in ESTS. Despite a positive margin in 72% of patients in our data, a complete response was reported in 59%.^2^ We believe that the negative impact of positive margin was effectively offset by adjuvant RT. Since sarcomas are considered relatively radioresistant tumors, we believe that the radiation doses of up to 50 Gy used in the neoadjuvant setting are somewhat suboptimal and higher doses of up to 66 Gy given postoperatively are more effective. The proponents of neoadjuvant RT recommend for a postsurgical RT boost of 16 Gy in cases with positive margins after preoperative RT, but splitting a course of RT results in losing the cumulative impact of biologically effective tumoricidal RT doses. There are data to suggest that postoperative RT boost for positive margins does not improve local control.^9^ Furthermore, RT or chemotherapy, either neoadjuvant or adjuvant, is not a substitute for definitive surgical resection with negative margins and every possible attempt should be considered for a resurgery to achieve negative margins without sacrificing functional outcome.

Our retrospective ESTS patient data are meant for identifying the gaps in our treatment process, based on which a better treatment strategy can be further designed. We agree that there is a need to identify a subset of patients who will benefit from chemotherapy and using nomograms is the way forward. We are still inclined more toward the use of RT in the adjuvant setting as evident by a complete response in 59% of patients in our study.^2^ Our team is grateful to Mangla for bringing this important issue forward via this correspondence. It is a very healthy academic discussion, and we will be happy to discuss it further, if required.

## References

[1] Mangla A Should neoadjuvant treatment be adopted more widely for patients with extremity soft tissue sarcoma in low-income countries? JCO Glob Oncol 10.1200/GO.23.00110 10.1200/GO.23.00110 PMC10581658 37441745

[2] Raza SM Riaz A Mansha MA et al Radiotherapy and limb-sparing surgery in the management of localized soft tissue sarcomas: Tertiary care center experience from Pakistan JCO Glob Oncol 10.1200/GO.22.00047 2023 10.1200/GO.22.00047 PMC10281447 36989464

[3] Abbasi AN Establishment and maintenance of quality of site-specific multidisciplinary tumor boards in Pakistan J Coll Physicians Surg Pak 26 805 807 2016 27806806

[4] Qureshi BM Mansha MA Karim MU et al Impact of peer review in the radiation treatment planning process: Experience of a Tertiary Care University Hospital in Pakistan J Glob Oncol 5 1 7 2019 10.1200/JGO.19.00039 PMC6733206 31393752

[5] Cormier JN Huang X Xing Y et al Cohort analysis of patients with localized, high-risk, extremity soft tissue sarcoma treated at two cancer centers: Chemotherapy-associated outcomes J Clin Oncol 22 4567 4574 2004 15542808 10.1200/JCO.2004.02.057

[6] Grobmyer SR Maki RG Demetri GD et al Neo-adjuvant chemotherapy for primary high-grade extremity soft tissue sarcoma Ann Oncol 15 1667 1672 2004 15520069 10.1093/annonc/mdh431

[7] Danieli M Gronchi A Staging systems and nomograms for soft tissue sarcoma Curr Oncol 30 3648 3671 2023 37185391 10.3390/curroncol30040278 PMC10137294

[8] Gronchi A Palmerini E Quagliuolo V et al Neoadjuvant chemotherapy in high-risk soft tissue sarcomas: Final results of a randomized trial from Italian (ISG), Spanish (GEIS), French (FSG), and Polish (PSG) sarcoma groups J Clin Oncol 38 2178 2186 2020 32421444 10.1200/JCO.19.03289

[9] Pan E Goldberg SI Chen YL et al Role of post-operative radiation boost for soft tissue sarcomas with positive margins following pre-operative radiation and surgery J Surg Oncol 110 817 822 2014 25111884 10.1002/jso.23741

